# Precise Detection of Wrist Pulse Using Digital Speckle Pattern Interferometry

**DOI:** 10.1155/2018/4187349

**Published:** 2018-06-07

**Authors:** Hengfei Zhang, Sijin Wu, Weixian Li, Yonghong Wang, Mingli Dong, Lianxiang Yang

**Affiliations:** ^1^School of Instrumentation Science and Opto-Electronics Engineering, Beijing Information Science and Technology University, Beijing 100192, China; ^2^School of Instrument Science and Opto-Electronics Engineering, Hefei University of Technology, Hefei, Anhui 23009, China; ^3^Department of Mechanical Engineering, Oakland University, Rochester, MI 48309, USA

## Abstract

Pulse diagnosis is one of the four diagnostic methods of traditional Chinese medicine. However it suffers from the lack of objective and efficient detection method. We propose a noncontact optical method to detect human wrist pulse, aiming at the precise determination of the temporal and spatial distributions of pulse. The method uses the spatial-carrier digital speckle pattern interferometry (DSPI) to measure the micro/nanoscale skin displacement dynamically. Significant improvements in DSPI measurement have been made to allow the DSPI to detect the comprehensive information of the arterial pulsation at locations of Cun, Guan, and Chi. The experimental results prove that the spatiotemporal distributions of pulse can be obtained by the proposed method. The obtained data can be further used to describe most of the pulse parameters such as rate, rhythm, depth, length, width, and contour.

## 1. Introduction

**Figure 1:**
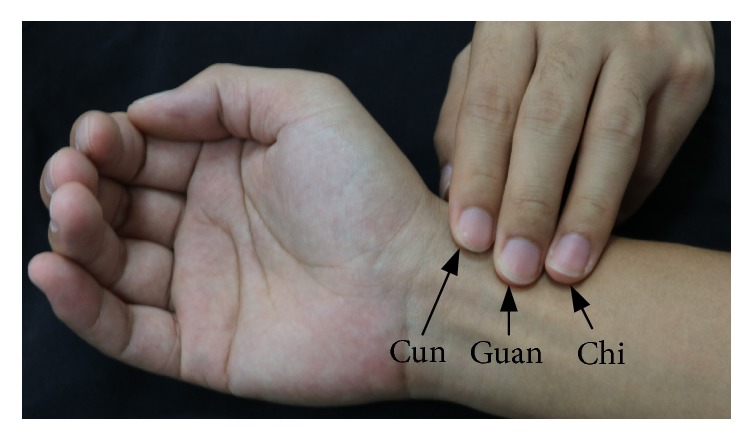
Pulsation at the locations of Cun, Guan, and Chi.

Human arterial pulsation is considered as a vital sign. It includes much information of human health. In Chinese traditional medicine (TCM), the arterial pulsation at the locations of Cun, Guan, and Chi, which are near wrist joint, as shown in Figure 1, is used for health evaluation and disease diagnosis. The method of wrist pulse investigation is called pulse diagnosis, one of the four diagnostic methods in TCM. The pulse diagnosis has played an important role during the past one thousand years in East Asia and also been a practical diagnostic method in modern society [1–3]. Many examples of health evaluation and disease diagnosis using the pulse diagnosis have been introduced [4–6]. The pulse diagnosis is also a practical method in other oriental medicines, such as Indian Ayurveda [7].

Traditionally, physicians of Chinese medicine touch patient's wrist and feel the pulsations at the three locations: Cun, Guan, and Chi. The diagnostic test relies entirely on the doctor's own experiential judgment. This subjective test may be affected by many factors. Moreover, the diagnostic results are hard to record and exchange. Thus, the quantification and standardization of the pulse diagnosis are very important. It requires the transformation of doctors' subjective feelings into objective physical quantities. To achieve this goal, the precise determination of the temporal and spatial distributions of the wrist pulse is required.

Owning to the development of modern transducers and computer technology, many physical methods have been presented to detect the wrist pulse during the last thirty years. These methods are mainly classified into pressure sensing and optical test methods. The pressure sensing methods are further divided into piezoresistive, piezoelectric, and piezocapacitive sensors. These sensors are attached to wrist skin tightly in order to sense the pulse pressure. For example, Sook Hyang Yoon et al. attached three semiconductor resistance strain gauge piezoresistive sensors at the three locations of Cun, Guan, and Chi to detect their pressure changes and pulse waves [8]. Kalange et al. used a single piezoelectric transducer to obtain the ideal pulse shape [7]. Hu developed a piezocapacitive sensor array that enabled multipoint detection of pulse [9]. Piezoelectric sensor based on polyvinylidene fluoride (PVDF) is a promising sensor for pulse detection due to its advantages of thin and soft structure, matching well with the acoustic impedance of human's soft tissue, and ease to achieve multipoint measurement [10]. Another promising material for pressure sensor is newly developed extraordinarily stretchable all carbon collaborative nanoarchitectures which can be used as an epidermal sensor [11]. The pressure sensing method is the dominating method in human pulse detection because of its highly sensitive detection and ease of use. However, the insufficient information of its test results makes it an unideal pulse detection method. Only a single or several points of artery are tested. The important information of the spatial distribution such as pulse length and width is lost. Moreover, though it is sensitive to arterial pulsation, it is hard to convert the outputs of the pressure transducers to the depth of wrist pulse directly. Consequently, the pressure sensing method has not been an ideal method of pulse detection yet.

Optical method can be divided into point measurement and full-field measurement methods. These methods detect the pulse by measuring the displacements of the human skin at selected locations or the full field. The optical point measurement, such as optical interferometry [12], laser triangulation [13], and optical fiber measurement [14], suffers from the same problem of the pressure sensing method that cannot get the spatial distribution of the wrist pulse.

Full-field measurement using optical method is desired to allow the obtainment of the pulse spatial distributions. Efforts in this research area mainly focus on vision measurement and digital image correlation (DIC) techniques. For example, Zhang developed an image device to satisfy the full-field detection of pulse [15]. Shao and Dai proposed a DIC method to realize the real-time and full-field measurement of pulse [16]. Xue and Su subsequently improve the pulse detection method using DIC and exhibit much reasonable results [17]. Both vision measurement and DIC can get the temporal and spatial distributions of wrist pulse, allowing much comprehensive information of wrist pulse being obtained. However, the measuring resolution is limited to several micrometers or even worse by their measuring principle. This is not enough to precisely determine the pulse amplitude at any locations. For example, the maximum pulse amplitudes at some locations beside Cun, Guan, and Chi are always less than several micrometers. It means the pulses at Cun, Guan, and Chi can be detected by these methods, but the pulses at other locations may not be successfully detected. In addition, additional cooperation target such as grid line and water transfer printing speckle patterns on the skin surface is required when vision measurement or DIC is used, reducing the convenience of pulse detection.

We introduce a precise pulse detection method based on digital speckle pattern interferometry (DSPI), allowing the highly sensitive detection of the temporal and spatial distributions of the pulse and the obtainment of more comprehensive and detailed pulse information. DSPI is a full-field optical technique which can measure the displacement/deformation distributions of object surfaces. Compared to vision measurement and DIC, DSPI enjoys significant advantages of much higher measuring accuracy and independence from cooperation target [18, 19]. As an optical interference technique, its resolution reaches 20 nm, which can trace back to the laser wavelength and is unsusceptible to device and external factors. Thus, slight change of the pulse can be detected by DSPI easily. We also improve the DSPI in data sampling, phase unwrapping, and data filtering, to achieve the in vivo detection of the human wrist pulse.

## 2. Methods

### 2.1. Principle and Experimental Setup of Spatial-Carrier DSPI

**Figure 2:**
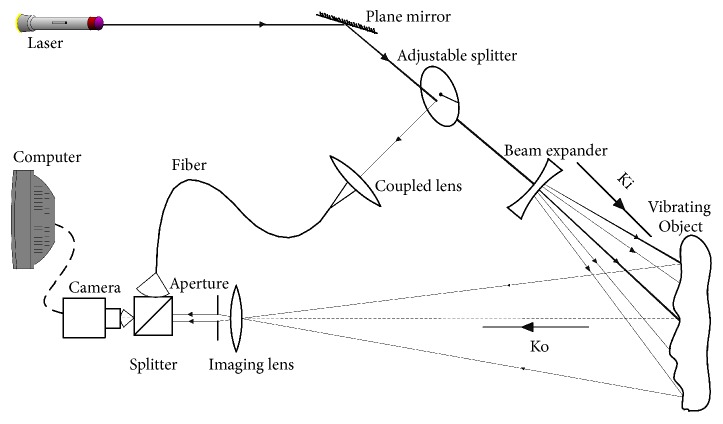
Optical setup for wrist pulse detection.

DSPI is classified, based on their optical setups and phase determination methods, into phase-shifting DSPI (PS-DSPI) and spatial-carrier DSPI (SC-DSPI) [20]. The SC-DSPI is also known as digital holographic interferometry [21]. The SC-DSPI outperforms the PS- DSPI in measuring speed, so it is a more competitive candidate for pulse detection. The optical arrangement of the SC-DSPI is depicted by Figure 2. The laser beam is divided into two beams, the object beam and reference beam, by an adjustable splitter. The object beam illuminates the vibrating object surface via a beam expander along the illumination direction* K*_*i*_. The scattered object beam is collected by an imaging lens along the observation direction* K*_*o*_ and then forms an image on the image sensor of the camera via an aperture. The reference beam is coupled into a fiber and then strikes the image sensor at a small angle between it and the optical axis. The object and reference beams encounter each other on the image sensor, yielding optical speckle interference.

The intensity distribution of the interferogram captured by the camera is given by

(1) Ix,y=Ox,y2+Rx,y2+Ox,yRx,y∗+Rx,yOx,y∗,

where *O* and *R* are the object and reference beams, respectively, (*x*, *y*) is the two-dimensional (2D) spatial coordinates on the image plane, and *∗* is the operator of complex conjugate.

The inclination angle of the reference beam towards the optical axis determines the spatial frequency of the speckle interferogram. The maximal spatial frequency is calculated by

(2) $$\begin{array}{c} f_{max}=\frac{\lambda }{2}\sin\left(\;\frac{\theta_{max}}{2}\;\right), \end{array}$$

where *λ* is the wavelength of the laser used in the experiment and *θ*_max_ is the maximal inclination angle of the reference beam. According to the Nyquist-Shannon sampling theorem, the experimental setup needs to meet the condition 2*f*_max_ < 1/*p*, where* p* is the size of the camera pixel. This condition limits the range of the reference beam inclination angle. Details about the determination of the reference beam's deflection can be found in [22].

### 2.2. Collection of Speckle Interferograms and Phase Reconstruction

The last two terms of (1) contain the information of the object beam's phase. These terms are separated from the background in the frequency domain by performing a 2D fast Fourier Transform (FFT) on the recorded interferogram *I*(*x*, *y*). Subsequently the complex amplitude of the object beam's wavefront is obtained after performing an inverse fast Fourier Transform (IFFT) on the selected frequency component. Due to the sensor discretization, the image captured by the camera composes m×n pixels whose sizes are Δ*x*×Δ*y*, where m and n are integer numbers. The speckle interferogram's intensity can be rewritten as *O*(*m*Δ*x*, *n*Δ*y*). The phase of the wavefront can be calculated by

(3) ΦwmΔx,nΔy=arctan⁡Im⁡OmΔx,nΔyRe⁡OmΔx,nΔy,

where the subscript *w* denotes a wrapped phase and Im and Re indicate the imaginary and real parts, respectively.

DSPI records a sequence of speckle interferograms and determines the phase Φ_*w*_(*m*Δ*x*, *n*Δ*y*, *i*Δ*τ*) at the point (*m*Δ*x*, *n*Δ*y*) and the time* i*Δ*τ*, where Δ*τ* is the time interval between two camera exposures. The phase difference between two adjacent phase distributions according to time (*i*-1) Δ*τ* and* i*Δ*τ* can be calculated by

**Figure 3:**
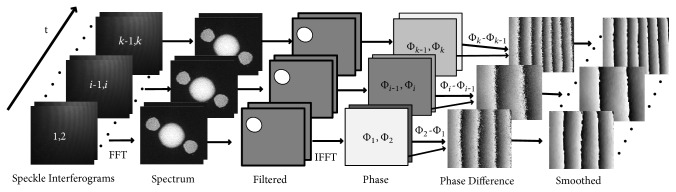
Process of the phase difference map calculation.

(4) ΔΦi,i−1=ΦwmΔx,nΔy,iΔτ−ΦwmΔx,nΔy,i−1Δτ,

After the phase differences are obtained, a sine/cosine average filter is used to filter the noise at the obtained phase maps, yielding smoothed phase maps. The entire process of digital speckle interferogram collection and phase reconstruction is shown in Figure 3.

The relationship between the phase difference and displacement** d** is given by

(5) $$\begin{array}{c} \Delta \Phi =\frac{2\pi }{\lambda }ds, \end{array}$$

where **s** = *K*_*i*_ − *K*_*o*_ is the sensitivity vector which is determined by the geometric layout of the measuring setup.

Usually the angle between the illumination and observation directions is very small. The absolute value of the sensitivity vector** s **is almost 2. Consequently, the out-of-plane deformation is calculated by

(6) $$\begin{array}{c} w_{i,i-1}=\frac{\lambda }{4\pi }\Delta \Phi_{i,i-1}, \end{array}$$

where **w**_*i*,*i*−1_ is the out-of-plane deformation during the time interval.

### 2.3. Phase Unwrapping

Phase unwrapping (PU) is a process to remove the 2*π* discontinuities within the phase image. It detects a 2*π* phase jump and adds or subtracts integer multiple of 2*π* to successive pixels following that phase jump based on a threshold mechanism. The existing algorithms can be classified into three categories, that is, temporal phase unwrapping (TPU), spatial phase unwrapping (SPU), and spatiotemporal three-dimensional phase unwrapping (STPU) [23].

**Figure 4:**
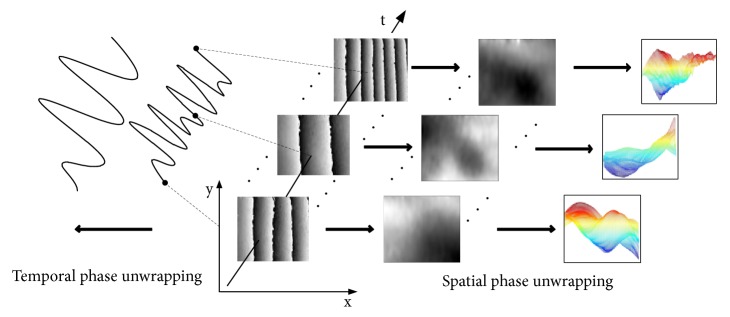
Procedure of spatiotemporal three-dimensional phase unwrapping.

TPU is carried out along the time axis due to the independence of each camera pixel. The phase at each pixel is treated as a function of time and unwrapped by a one-dimensional unwrapping algorithm. SPU involves a spatial comparison of phase values at neighboring pixels, which can be further classified into path-dependent and path-independent methods. STPU unwraps the whole phase map along the 2D spatial domain and several selected points along the time axis. The combination of the spatial and temporal phase distributions yields the dynamic phase difference maps. The operating procedure of STPU is depicted by Figure 4.

## 3. Experiment and Result Discussion

A 5 W diode pumped solid state laser with a central wavelength of 532nm (Coherent Verdi G5) and CMOS camera (CatchBEST MU3S230 (SGYYO)) with a frame rate of 80 frames per second (fps) at 2.3 megapixels were used in the experiment. The frame rate can be enhanced when the pixel resolution is reduced. The camera was triggered by an external clock signal which guaranteed the uniformly sampling of the wrist pulse. A nonspherical lens whose focal length is 100 mm was used for imaging.

Reference [16] shows that the maximum pulse displacement velocity is about 10 *μ*m/s. This velocity is transferred to the phase change rate of about 37.6 cycles per second according to (6). In order to satisfy the Nyquist-Shannon sampling theorem, at least 2 points per phase cycle should be sampled. Actually, at least 4 sample points per phase cycles should be provided to avoid the influence of phase noise. Thus, a sample rate of 150.4 Hz is necessary to satisfy the wrist pulse detection. In the experiment, the frame rate was set as 160 fps which is faster than the minimum requirement of sample rate, guaranteeing the effective sampling. This frame rate was realized by reducing the image region of interesting to 1500×500 pixels, corresponding to a measurement area of 75 mm × 25 mm.

**Figure 5:**
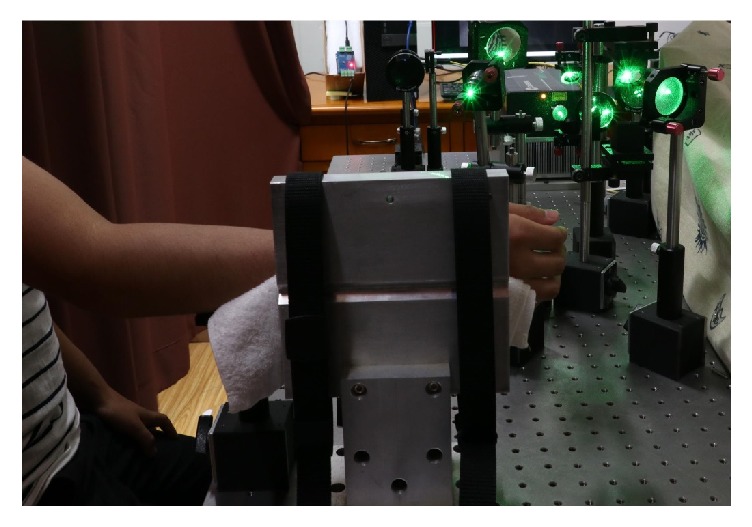
Experimental setup for wrist pulse detection.

The right wrist of a volunteer was fixed by two lashing bands on a backplane. In addition, the output of the laser was adjusted to 0.25 W to avoid human's discomfort. In the experiment, totally 28800 digital speckle interferograms were captured for further processing. The vibration of pulse during the test time of 3 minutes was then obtained. The experimental setup is shown in Figure 5.

**Figure 6:**
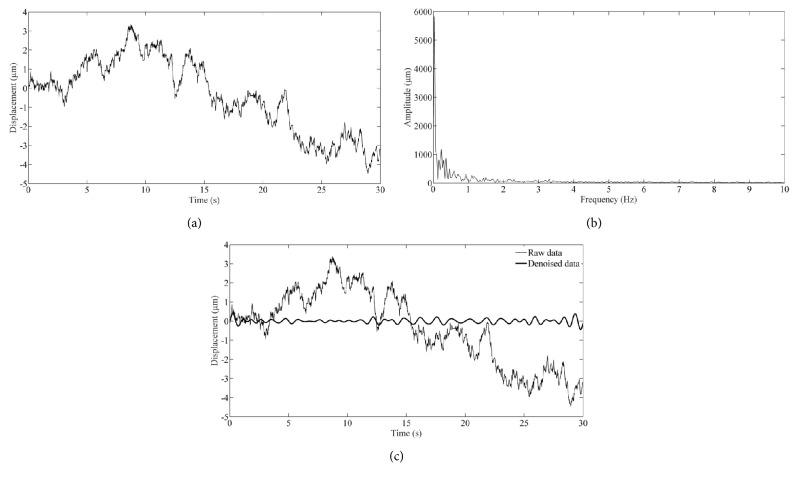
Absolute displacement of pulse at the location of pixel (290, 480): (a) raw data obtained by performing the temporal phase unwrapping; (b) amplitude spectrum; (c) raw and denoised data.

Results within a period of 30 s were selected for exhibition.  Figure 6(a) illustrates the absolute displacement variation at the location of pixel (290, 480). The complex and irregular data indicate the influence of a composite factor that includes the rigid body motion of human wrist and random displacement fluctuation of human skin. The quality of the result was much improved by applying a 0.7 Hz to 2 Hz band pass filtering towards the amplitude spectrum which is obtained by performing a FFT on the raw data. The amplitude spectrum and denoised data are shown in Figures 6(b) and 6(c), respectively.

**Figure 7:**
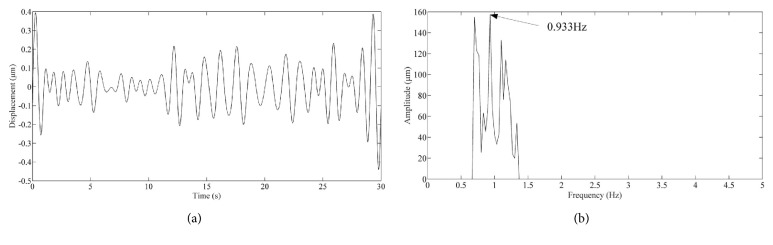
Detail of the pulse amplitude and frequency: (a) pulse displacement; (b) pulse amplitude spectrum.

The displacement detail and frequency spectrum of the pulse vibration are depicted by Figure 7.  Figure 7(a) shows the peak to peak value of the pulse amplitude is approximately 9 *μ*m at the selected location.  Figure 7(b) shows that the pulse frequency is 0.933 Hz which is equivalent to 60 pulses per minute.

**Figure 8:**
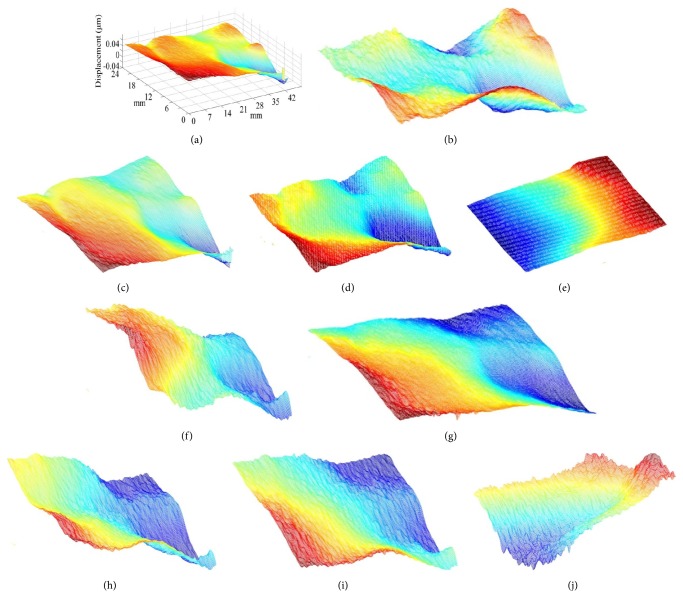
Spatial displacement distributions of the pulse.

The spatial distributions of pulse vibration are given by Figure 8. These displacement maps correspond to different time of the pulse vibration.

## 4. Conclusions

The paper presents a human wrist arterial pulsation detection method using SC-DSPI. The feasibility of pulse detection using the presented method is illustrated by the theoretical analysis as well as the experiment. The advantage of noninvasive, full-field, and high-precision detection has also been demonstrated.

## References

[1] Jeon Y. J., Kim J. Y., Lee H. J. (2011). A clinical study of the pulse wave characteristics at the three pulse diagnosis positions of Chon, Gwan and Cheok. *Evidence-Based Complementary and Alternative Medicine*.

[2] Dharmananda S. (2004). *The significance of traditional pulse diagnosis in the modern practice of Chinese medicine*.

[3] Tang A. C. Y., Chung J. W. Y., Wong T. K. S. (2012). Validation of a novel traditional Chinese medicine pulse diagnostic model using an artificial neural network. *Evidence-based complementary and alternative medicine*.

[4] Zhang J., Wang N., Liu J., Zhou L., Lei Y. (2017). Research of features in the pulse waves of balanced constitution women during the second trimester and third trimester of pregnancy. *China Journal of Traditional Chinese Medicine & Pharmacy*.

[5] Shin K. Y., Lee T. B., Jin S. O. Characteristics of the pulse wave in patients with chronic gastritis and the healthy in Korean Medicine.

[6] Zhang H., Yu Z., Fu L. (2017). Objective evaluation of acupuncture treatment in patients with cervical spondylosis by pulse diagnosis device. *BIO Web of Conferences*.

[7] Kalange A. E., Gangal S. A. (2007). Piezoelectric sensor for human pulse detection. *Defence Science Journal*.

[8] Yoon S. H., Koga Y., Matsumoto I., Ikezono E. (1987). An objective method of pulse diagnosis.. *American Journal of Chinese Medicine*.

[9] Hu C., Chung Y., Yeh C., Luo C. (2012). Temporal and Spatial Properties of Arterial Pulsation Measurement Using Pressure Sensor Array. *Evidence-Based Complementary and Alternative Medicine*.

[10] Jin G., Yu M., Bao N. (1999). Research on PVDF multipoint pulse wave computer aided test system. *Chinese Journal of J Tsinghua University (Scice & Technology)*.

[11] Cai Y., Shen J., Dai Z. (2017). Extraordinarily Stretchable All-Carbon Collaborative Nanoarchitectures for Epidermal Sensors. *Advanced Materials*.

[12] Hong H., Fox M. D. (1994). No Touch Pulse Measurement by Optical Interferometry. *IEEE Transactions on Biomedical Engineering*.

[13] Wu J.-H., Chang R.-S., Jiang J.-A. (2007). A novel pulse measurement system by using laser triangulation and a CMOS image sensor. *Sensors*.

[14] Li S., Zhang F., Ni J., Wang C., Wang H. (2016). Point-contact type FBG dynamic pressure sensor and its application in the measurement of pulse information. *Journal of Optoelectronics Laser*.

[15] Zhang A., Yang L., Dang H. (2014). Detection of the typical pulse condition on Cun-Guan-Chi based on image sensor. *Sensors & Transducers Journal*.

[16] Shao X., Dai X., Chen Z., He X. (2016). Real-time 3D digital image correlation method and its application in human pulse monitoring. *Applied Optics*.

[17] Xue Y., Su Y., Zhang C. (2017). Full-field wrist pulse signal acquisition and analysis by 3D Digital Image Correlation. *Optics & Lasers in Engineering*.

[18] Yang L., Xie X., Zhu L., Wu S., Wang Y. (2014). Review of electronic speckle pattern interferometry (ESPI) for three dimensional displacement measurement. *Chinese Journal of Mechanical Engineering*.

[19] Wang Y., Sun J., Li J., Gao X., Wu S., Yang L. (2016). Synchronous measurement of three-dimensional deformations by multicamera digital speckle patterns interferometry. *Optical Engineering*.

[20] Wu S., Dong M., Fang Y., Yang L. (2016). Universal optical setup for phase-shifting and spatial-carrier digital speckle pattern interferometry. *Journal of the European Optical Society: Rapid Publications*.

[21] Pedrini G., Osten W., Gusev M. E. (2006). High-speed digital holographic interferometry for vibration measurement. *Applied Optics*.

[22] Liu K., Wu S., Gao X., Zhu L., Yang L. (2015). Research on the Key Parameters of Optical Path for Spatial-carrier Digital Speckle Pattern Interference. *Chinese Journal of Process Automation Instrumentation*.

[23] Wu S., Zhu L., Pan S., Yang L. (2016). Spatiotemporal three-dimensional phase unwrapping in digital speckle pattern interferometry. *Optics Expresss*.

